# Therapeutic Targeting of GSK3β-Regulated Nrf2 and NFκB Signaling Pathways by Salvianolic Acid A Ameliorates Peritoneal Fibrosis

**DOI:** 10.3389/fmed.2022.804899

**Published:** 2022-03-07

**Authors:** Fan Zhou, Lan Yao, Xiaoqing Lu, Yubao Li, Xingmin Han, Pei Wang

**Affiliations:** ^1^Department of Nuclear Medicine, The First Affiliated Hospital of Zhengzhou University, Zhengzhou, China; ^2^Henan Medical Key Laboratory of Molecular Imaging, Zhengzhou, China; ^3^Blood Purification Center, Institute of Nephrology, The First Affiliated Hospital of Zhengzhou University, Zhengzhou, China

**Keywords:** peritoneal dialysis, epithelial cells, oxidative stress, inflammation, fibrosis, herb, *Salvia miltiorrhiza*

## Abstract

Peritoneal fibrosis is a devastating complication in patients undergoing peritoneal dialysis, with no definite therapy yet available. *Salvia miltiorrhiza* and its major active component Salvianolic acid A (Sal A) have demonstrated a beneficial effect in myriad diseases. However, their effect on peritoneal fibrosis is unknown. In murine models of peritoneal dialysis, daily Sal A treatment substantially improved the peritoneal dialysis fluid (PDF) elicited peritoneal fibrosis, marked by thickening of the submesothelial compact zone, accumulation of extracellular matrix and increased expression of vimentin and PAI-1, concomitant with attenuation of GSK3β hyperactivity. This coincided with diminished nitrotyrosine in peritoneal tissues and increased Nrf2 nuclear translocation, entailing a lessened oxidative injury and reinforced Nrf2 antioxidant response. Meanwhile, inflammatory infiltration and maladaptive angiogenesis in peritoneal tissues provoked by PDF injury were also mitigated by Sal A, associated with a suppressed NFκB activation. Mechanistically, ectopic expression of the constitutively active GSK3β blunted the NFκB-suppressing and Nrf2-activating efficacy of Sal A in peritoneal mesothelial cells exposed to hypertonic dextrose, suggesting that GSK3β inhibition mediates the protective effect of Sal A. Collectively, our findings may open the avenue for developing a novel therapy based on Sal A for preventing peritoneal fibrosis in peritoneal dialysis.

## Introduction

Peritoneal dialysis is one of the very few renal replacement therapeutic modalities available to patients with end-stage renal failure or uremia (1). The efficacy of peritoneal dialysis is highly dependent on the functionality of the peritoneal membrane that lines over the abdominal cavity of the peritoneum and serves as a semipermeable membrane, through which body water and metabolic wastes passes into the dialysis fluid until equilibrium (2). The peritoneal membrane is comprised mainly by the peritoneal mesothelial cells, which may dedifferentiate and acquire profibrogenic phenotypes upon long term exposure to a number of injurious factors, including sustained hypertonicity of peritoneal dialysis solutions, uremic toxins, peritonitis, infection and inflammatory mediators, ultimately culminating in peritoneal fibrosis and peritoneal membrane ultrafiltration dysfunction, with no definite therapy yet available (1, 2). In order to sustain a critical life-line for uremic patients, it is imperative to identify novel therapeutic targets and develop new treatments for preventing peritoneal fibrosis.

Akin to fibrosis in other organ systems (3), peritoneal fibrosis is a complex yet well-orchestrated pathological process and involves oxidative stress, inflammation, maladaptive angiogenesis and other events (1). A number of cellular signaling pathways have been implicated in this process. Among these, the nuclear factor-erythroid factor 2-related factor 2 (Nrf2) antioxidant response has been regarded as the master mechanism for self-defense against oxidative injury (4). In addition, the NFκB pathway plays a key role in inflammatory response and maladaptive angiogenic endothelial proliferation (5). Recent studies demonstrate that GSK3β is situated at the nexus of the Nrf2 and NFκB pathways and serves as a crucial regulator of the above pathological events (6, 7). Indeed, burgeoning evidence has implicated GSK3β in the fibrogenesis of diverse tissues (8, 9), including peritoneal mesothelial cells (10). As such, therapeutic targeting of GSK3β is likely a promising strategy to treat peritoneal fibrosis.

**Figure 1:**
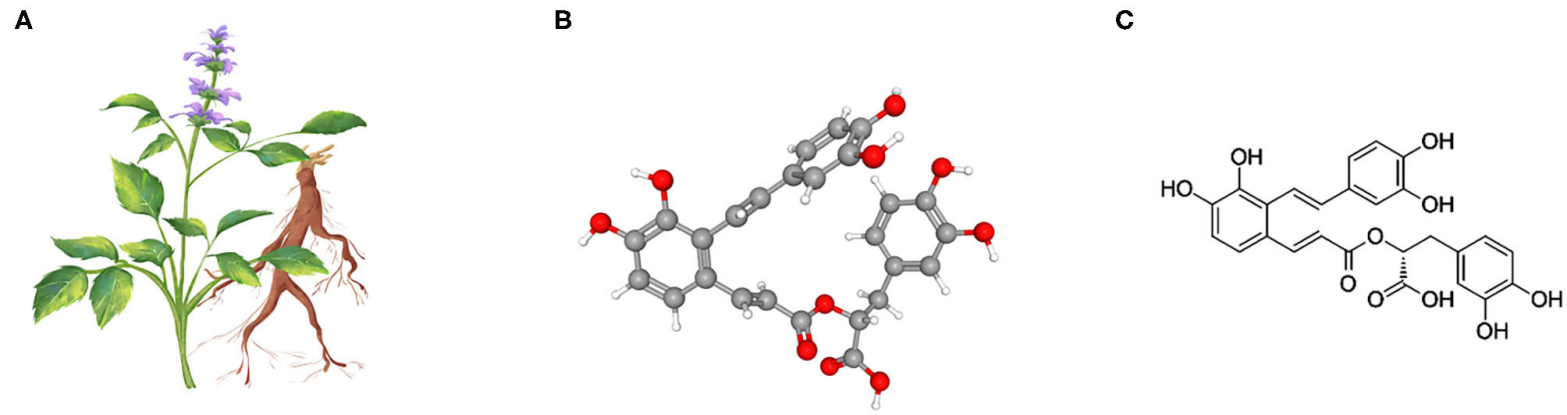
*Salvia miltiorrhiza* and the chemical structure of its key active component Salvianolic acid A (Sal A). **(A)** Illustration of *Salvia miltiorrhiza* and the medicinal root; **(B)** As the most abundant water-soluble active component extracted from *Salvia miltiorrhiza*, Sal A is a rosmarinic acid derivative and a member of the class of 1-benzofurans. The 3-D view of the conformer of Sal A; **(C)** The 2-D view of the chemical structure of Sal A.

*Salvia miltiorrhiza*, also known as red sage, is an herb widely used in traditional Chinese medicine to treat cardiovascular and cerebrovascular disease (11, 12). A growing body of evidence recently suggests that *S. miltiorrhiza* is likely to confer a multi-pronged beneficial effect in various organ systems *via* a potent antioxidant and anti-inflammatory mechanism (11). The main effective molecules are still uncertain, but there is evidence suggesting that *S. miltiorrhiza* extractants contain high amounts of Salvianolic acid A (Sal A), a rosmarinic acid derivative and a member of the class of 1-benzofurans (Figure 1) and an antioxidant, anti-inflammatory, hypoglycemic, hepatoprotective, neuroprotective, cardioprotective agent (11). Mechanistically, Sal A is able to perturb a number of cell signaling pathways related to fibrogenesis, such as GSK3β (13, 14). Recent studies demonstrate a potent anti-fibrotic effect of Salvianolic acid A in multiple organs (15), including the kidney (13), liver (16), and lung (17). However, the effect of *S. miltiorrhiza* or its effective component Sal A on peritoneal fibrosis has not been studied before. This study aimed to examine the possible effect of Sal A on peritoneal fibrosis *in vivo* in murine models of peritoneal dialysis and *in vitro* in cultured peritoneal mesothelial cells.

## Materials and Methods

### Animal Study Design

Male B6 mice (8–10-weeks old) were used in this study. The mouse model of peritoneal fibrosis was established by daily intraperitoneal injection of 3 ml of 4.25% dextrose peritoneal dialysis fluid (PDF, Baxter HealthCare, Deerfield, IL, USA) as previously described (18). Mice treated with the same volume of PBS served as normal control. PDF or PBS-treated mice were randomized to receive daily intraperitoneal injection of vehicle or Sal A (60 mg/kg, Sigma, MO, USA) according to the dose used before (19). All animals were euthanized on week 7 and peritoneal tissues including the anterior abdominal wall were collected for further examination. All experimental procedures were approved by the Ethics Committee at the First Affiliated Hospital of Zhengzhou University.

### Cell Cultures

Primary mouse peritoneal mesothelial cells (PMC) were isolated by enzymatic digestion of the inner surface of the peritoneum and then cultured in culture medium containing DMEM with 20% FBS as described previously (20). In brief, immediately after euthanasia, 10 ml of 0.25% tripsin solution was injected into the peritoneum of mice. After 10 min, the trypsin solutions were collected with needle and syringe and the peritoneal cavities were rinsed with culture medium, which was collected and transferred together with the trypsin solutions to a centrifuge tube and centrifuged. The cell pellet was collected and re-suspended in culture medium. The cells were characterized to possess typical phenotypes of mesothelial cells, including cytokeratin and calretinin with negligible contamination by leukocytes. Cells were subjected to Lipofectamine-transient transfection with the empty vector or the vector encoding the hemagglutinin (HA)-conjugated constitutively-active mutant of GSK3β as previously elaborated (21). 24 h after transfection, cells were then pretreated with Sal A (50 μM) as reported before (22) or vehicle for 30 min prior to treatment with 4.25% dextrose or equal volume of PBS for 48 h.

### Western Immunoblot Analysis

Peritoneal tissues or collected cells were lysed in radioimmunoprecipitation assay buffer supplemented with protease inhibitors. NE-PER kit (Thermo Scientific, Rockford, Illinois, USA) was used to prepare the nuclear fractions according to the manufacturer's instruction. Protein samples were processed for immunoblot analysis as previously described (23). The antibodies against fibronectin, vimentin, PAI-1, nitrotyrosine, Histone H3, and β-actin were purchased from Santa Cruz Biotechnology (CA, USA). The antibodies against p-GSK3β, GSK3β, p-NFκB, NFκB, HA, and β-tubulin were purchased from Cell Signaling Technology (MA, USA). The antibody against Nrf2 was acquired from Abcam (CA, USA).

### ELISA Analyses

Peritoneal lysates were subjected to Enzyme-linked immunosorbent assay (ELISA) for measuring the expression levels of TNF-α, IL-1β, MCP-1 protein in accordance with the manufacturer's instructions (R&D Systems, MN, USA).

### Histology and Immunostaining

Formalin-fixed paraffin-embedded peritoneal tissues were processed for sections and following subjected to immunofluorescence staining or Masson's trichrome staining to assess histological changes and peritoneum thickness. The peritoneum sections were deparaffinized and rehydrated, heated in citrate buffer by microwave, quenched with 3% H_2_O_2_, blocked by 5% BSA, and incubated with the primary antibodies against the following molecules: CD45 (Santa Cruz Biotechnology), CD68 (Abcam), CD31 (Abcam), vascular endothelial growth factor (VEGF, Santa Cruz Biotechnology), nitrotyrosine (Santa Cruz Biotechnology), Nrf2 (Abcam), followed by staining with Alexa Fluor-conjugated secondary antibody (Life Technologies, Carlsbad, CA, USA). Finally, sections were mounted with mounting medium with 4′,6-diamidino-2-phenylindole (DAPI, Abcam), and visualized using a fluorescence microscope.

### Computerized Morphometric Analysis

The thickness of submesothelial compact zone between basal border of surface mesothelial cells and upper border of peritoneal adipose tissue was estimated by computerized morphometric analysis of Masson's trichrome-stained micrographs with 200× original magnification using the ImageJ analysis program, version 1.52a (National Institutes of Health, Bethesda, MD, USA). Five portions were randomly selected for the measurement and calculation of the average thickness of the submesothelial compact zone for each sample based on the five-point measurement method reported by Honda et al. (24).

### Statistical Analyses

The integrated pixel density of immunoblot bands was determined using a densitometer and the ImageJ analysis program. All *in vitro* studies were repeated at least three times. All data are expressed as mean ± SD. Differences in means were tested in Graphpad Prism 8 or SPSS 24. One-way ANOVA tests were performed for multiple comparisons of values followed by *Tukey's post-hoc* test. *P* < 0.05 was considered to represent a statistically significant difference.

## Results

### Peritoneal Fibrosis in the Mouse Model of Peritoneal Dialysis Is Improved by Sal A Treatment, Concomitant With the Attenuation of GSK3β Hyperactivity

**Figure 2:**
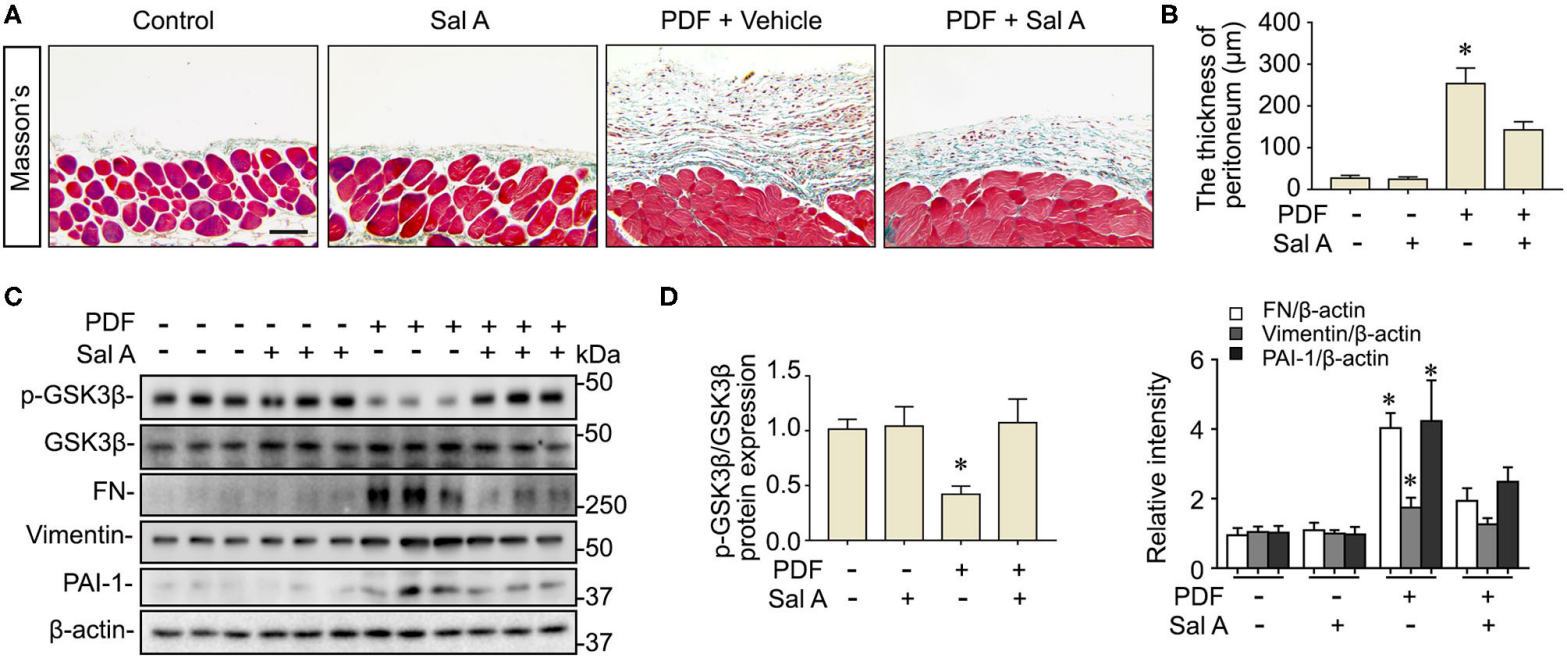
Sal A treatment ameliorates peritoneal fibrosis and averts GSK3β hyperactivity in the murine model of peritoneal dialysis. **(A)** Mice were randomized to receive daily intraperitoneal injection of 3 ml of 4.25% dextrose PDF or PBS in the presence of daily intraperitoneal injection of Sal A or vehicle. The peritoneal tissues were collected 7 weeks later and subjected to Masson's trichrome staining. Scale bar = 100 μm. **(B)** The thickness of the submesothelial compact zone was estimated by computerized morphometric analysis and data were expressed as mean ± SD and were analyzed by one-way ANOVA followed by Tukey's test. *P < 0.05 vs. all other groups (*n* = 6). **(C)** Representative immunoblots showing immunoblot analysis of peritoneal tissues for indicated molecules. **(D)** Relative abundance of indicated proteins, as normalized to the expression of β-actin or total GSK3β based on densitometric analyses of immunoblots (*n* = 6). Data were analyzed by one-way ANOVA followed by Tukey's test. *P < 0.05 vs. other groups.

Long-term stimulation by PDF has been associated with peritoneal fibrosis. Indeed, peritoneal fibrosis was evident in the mouse model of peritoneal dialysis on week 7, as revealed by Masson trichrome staining for collagens (Figure 2A). Computerized morphometric analysis demonstrated a marked thickening of the submesothelial compact zone as compared with PBS-treated mice (Figure 2B). Morphologic findings were corroborated by immunoblot analysis for peritoneal tissues, which demonstrated an increased expression of fibronectin, a key component of the extracellular matrix, vimentin, the intermediate filament and mesenchymal marker, and the profibrotic cytokine PAI-1 (Figures 2C,D). Sal A treatment significantly diminished collagen deposition in peritoneal tissues as shown by Masson's Trichrome staining, lessened the thickness of the submesothelial compact zone, and mitigated the PDF induced overexpression of fibronectin, vimentin and PAI-1 in peritoneal tissues as shown by immunoblot analysis (Figure 2). GSK3β has been implicated in tissue fibrogenesis in diverse organ systems. In consistency, the above findings of PDF-elicited peritoneal fibrosis were associated with GSK3β hyperactivity, marked by increased GSK3β expression and reduced inhibitory phosphorylation of GSK3β at serine 9 residue, as revealed by immunoblot analysis of peritoneal tissues (Figures 2C,D). In contrast, the PDF-triggered GSK3β hyperactivity was averted after Sal A, concomitant with the protective effect on peritoneal fibrosis.

### Sal A Treatment Reinforces the Nrf2 Antioxidant Response and Diminishes Peritoneal Oxidative Injury

**Figure 3:**
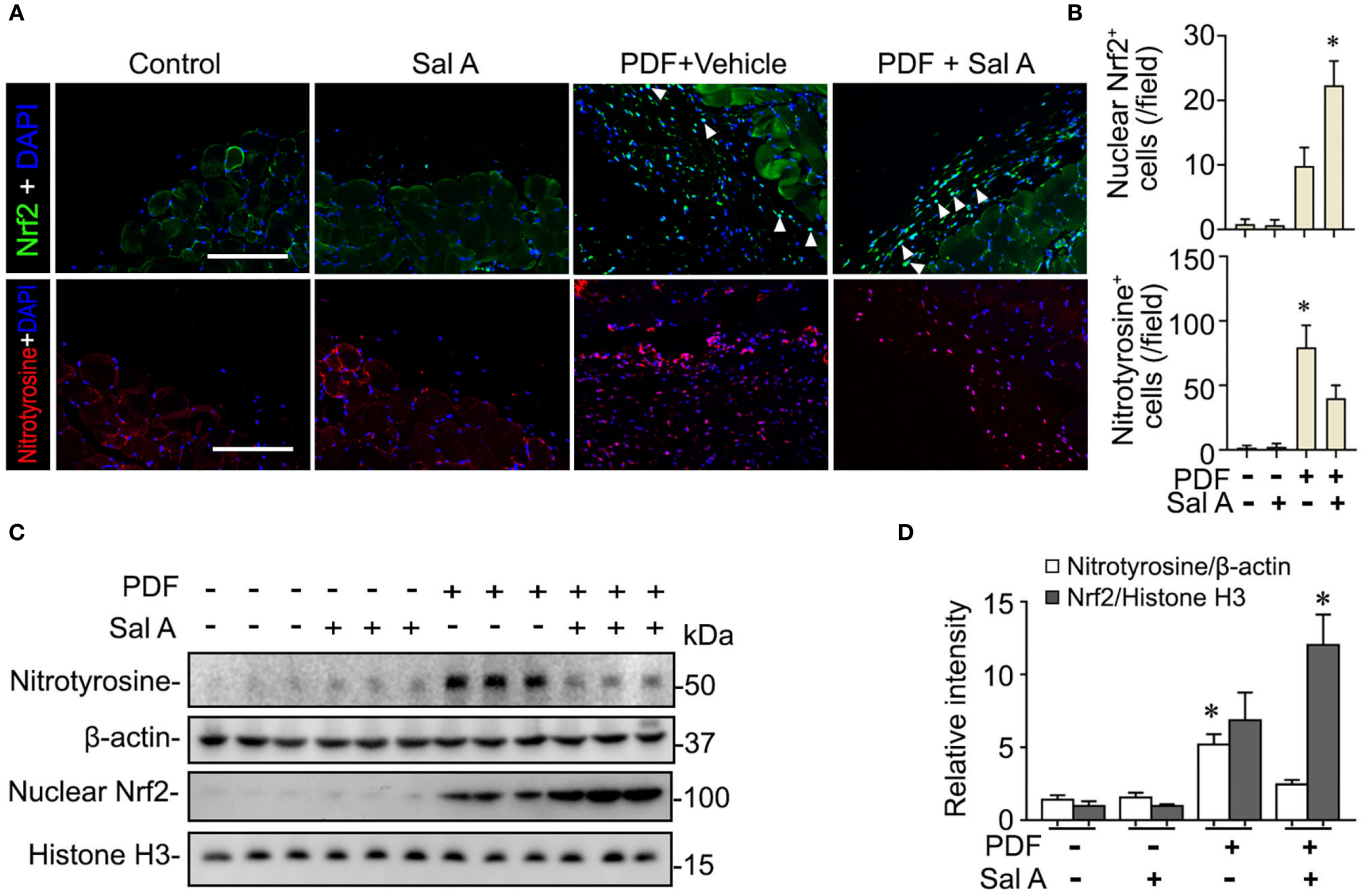
Sal A treatment of murine models of peritoneal dialysis reinforces Nrf2 antioxidant response and diminishes oxidative damage in peritoneum. **(A)** Mice were treated as elaborated in Figure 2. The peritoneal tissues were collected on week 7 and subjected to fluorescence immunohistochemistry staining for indicated proteins. The arrowheads indicate nuclear Nrf2 positive cells. Scale bar = 200 μm. **(B)** Absolute counting of nuclear Nrf2 or nitrotyrosine positive (+) cells. Data were analyzed by one-way ANOVA followed by Tukey's test. *P < 0.05 vs. other groups (*n* = 6). **(C)** Representative immunoblots showing immunoblot analysis of peritoneal tissues for indicated molecules. **(D)** Relative abundance of indicated proteins, as normalized to that of β-actin or histone based on densitometric analyses of immunoblots (*n* = 6). Data were analyzed by one-way ANOVA followed by Tukey's test. *P < 0.05 vs. other groups.

PDF is known to be able to instigate oxidative stress *via* various mechanisms such as hypertonicity. To ascertain if this resulted in any oxidative injury in the peritoneal tissues, fluorescent immunohistochemistry staining was performed. Shown in Figures 3A,B, PDF treatment caused a drastic induction of nitrotyrosine, a reliable *in vivo* marker of oxidative stress associated protein modification and dysfunction. Sal A treatment significantly reduced the expression of nitrotyrosine in PDF-stimulated peritoneum, denoting an antioxidant and protective activity. The histologic findings were consistently corroborated by immunoblot analysis of peritoneal tissues for nitrotyrosine combined with densitometry (Figure 3C). Upon oxidative stress, Nrf2 signaling will be immediately ignited as the master regulator of anti-oxidative responses and the primary cellular defense signaling against the cytotoxic effects of reactive oxygen species (ROS) and reactive nitrogen oxide species (RNOS). To determine if the Sal A conferred beneficial action on peritoneal oxidative injury stems from a possible effect on the Nrf2 antioxidant response, peritoneal tissues were subjected to fluorescent immunohistochemistry for Nrf2 (Figure 3A). Under basal conditions, Nrf2 staining was weak and mostly located to cytoplasm in peritoneal tissues. Upon PDF stimulation, there was a remarkable induction of Nrf2 response as a self-defense mechanism, shown by increased Nrf2 expression with some nuclear staining denoting nuclear translocation. Sal A treatment greatly enhanced Nrf2 antioxidant response, marked by reinforced Nrf2 nuclear translocation (Figures 3A,B). To quantify the histologic findings, nuclear fractions of peritoneal tissues were prepared and subjected to immunoblot analysis in combination with densitometry, which consistently demonstrated that nuclear expression of Nrf2 in PDF-treated peritoneal tissues was significantly potentiated by Sal A (Figures 3C,D).

### NFκB Activation in Peritoneal Tissues of Mouse Models of Peritoneal Dialysis Is Mitigated by Sal A, Resulting in Improved Peritoneal Inflammation, and Maladaptive Angiogenesis

**Figure 4:**
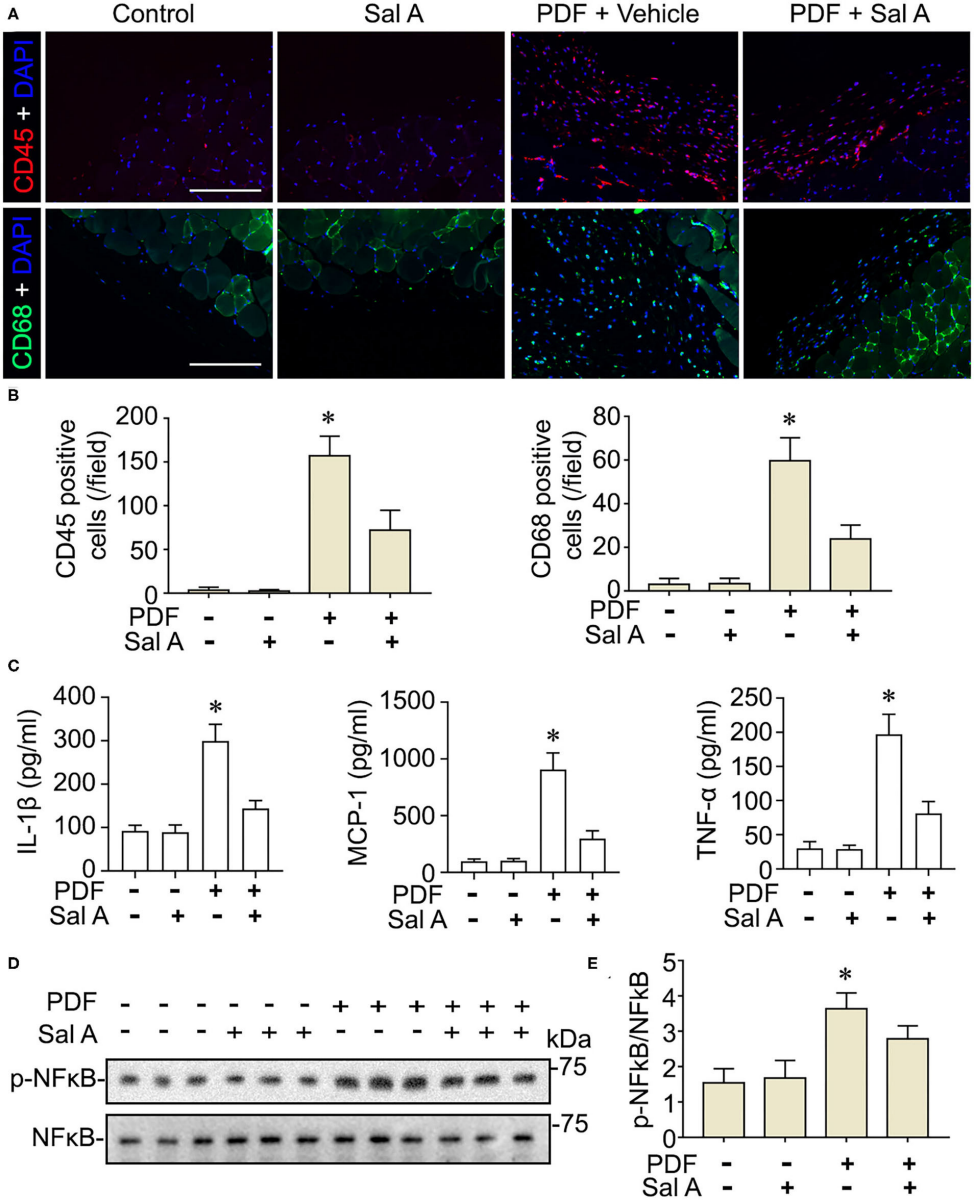
Sal A treatment of murine models of peritoneal dialysis attenuates inflammation and NFκB activation in peritoneum. **(A)** Mice were treated as elaborated in Figure 2. The peritoneal tissues were collected on week 7 and subjected to immunofluoresence staining for CD45 or CD68 with nuclear counterstaining of DAPI. Scale bar = 200 μm. **(B)** Absolute counting of CD45 or CD68 positive cells. Data were analyzed by one-way ANOVA followed by Tukey's test. *P < 0.05 vs. other groups (*n* = 6). **(C)** The expression levels of IL-1β, MCP-1, and TNF-α (*n* = 4). Data were analyzed by one-way ANOVA followed by Tukey's test. *P < 0.05 vs. other groups. **(D)** Representative immunoblots showing immunoblot analysis of peritoneal tissues for indicated molecules. **(E)** Relative abundance of p-NFκB p65, as normalized to that of NFκB p65 based on densitometric analyses of immunoblots (*n* = 6). Data were analyzed by one-way ANOVA followed by Tukey's test. *P < 0.05 vs. other groups.

**Figure 5:**
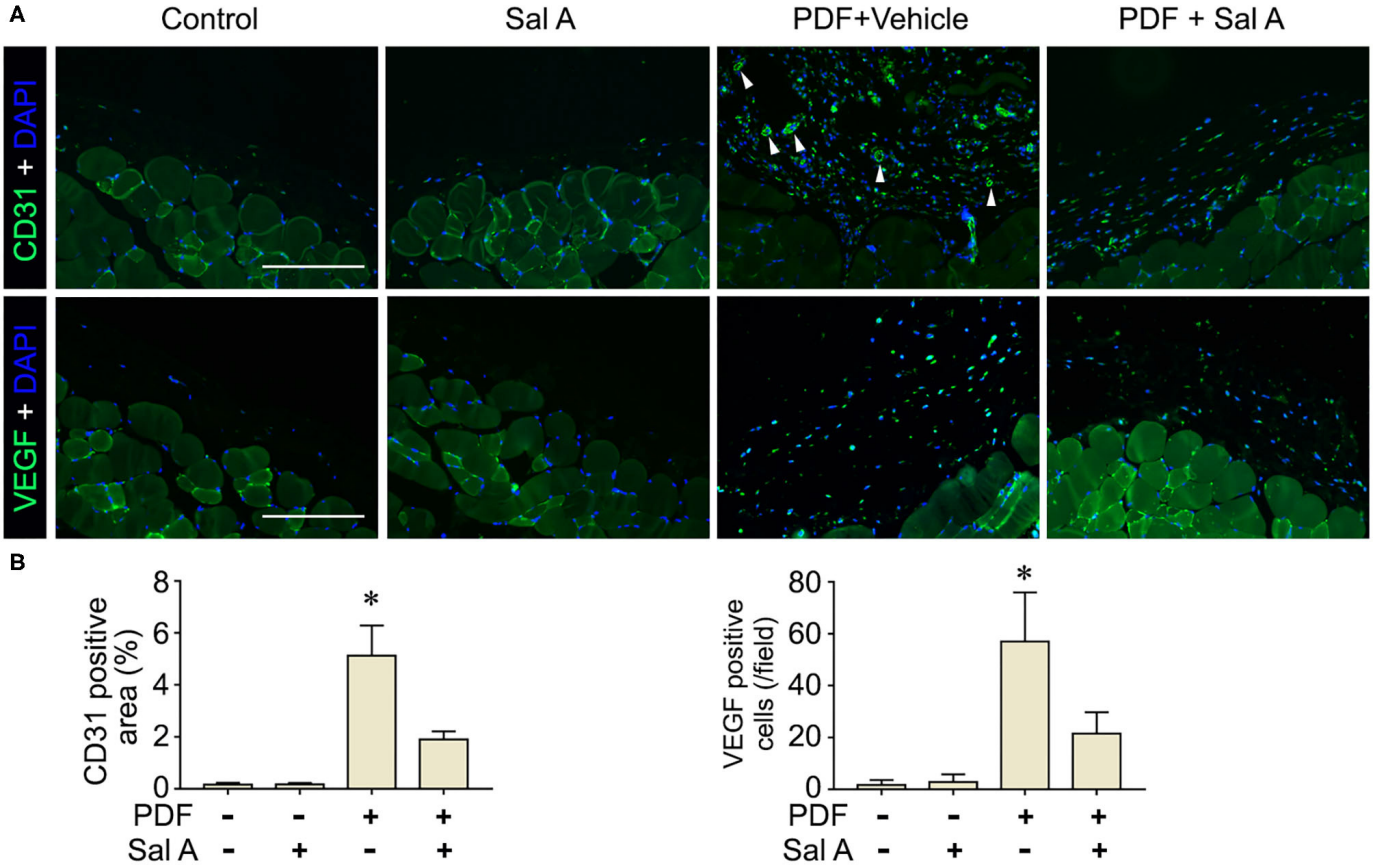
Sal A treatment of murine models of peritoneal dialysis mitigated endothelial proliferation and maladaptive angiogenesis in peritoneum. **(A)** Mice were treated as elaborated in Figure 2. The peritoneal tissues were collected on week 7 and subjected to fluorescence immunohistochemistry staining for CD31 and VEGF with nuclear counterstaining of DAPI. The arrowheads indicate CD31 positive vessels. Scale bar = 200 μm. **(B)** Quantification analysis of CD31 positive area and VEGF positive cells per microscopic field. Data were analyzed by one-way ANOVA followed by Tukey's test. *P < 0.05 vs. other groups (*n* = 6).

In addition to oxidative injury, PDF is also known to elicit peritoneal inflammation, which may be both the cause and consequence of peritoneal fibrosis. Indeed, fluorescent immunohistochemistry for CD45, the leukocyte common antigen, and CD68, a specific marker for monocytes/macrophages, revealed prominent inflammatory infiltration in peritoneal tissues stimulated by PDF (Figure 4A). This effect was markedly suppressed by Sal A treatment. The morphologic findings were further confirmed by absolute counting of CD45 and CD68 positive cells (Figure 4B). In consistency, the proinflammatory cytokines induced by PDF stimulation like TNF-α, IL-1β, and MCP-1 were significantly suppressed by Sal A treatment, as measured by ELISA (Figure 4C). Central to the pathogenic mechanisms of inflammation, NFκB pathway plays a key role. As shown by immunoblot analysis of peritoneal tissues followed by densitometry (Figures 4D,E), activation of NFκB, marked by phosphorylation of NFκB RelA/p65, was triggered by PDF stimulation, and this effect was substantially abrogated after Sal A treatment. As a pathological event closely associated with inflammation and NFκB activation, maladaptive angiogenesis also plays an instrumental role in peritoneal fibrosis. As shown by fluorescent immunohistochemistry for CD31, a standard marker for endothelial cells, PDF injury resulted in evident increase of the amount of endothelial cells in peritoneal tissues, denoting maladaptive angiogenesis (Figure 5A). This effect was considerably suppressed by Sal A treatment. Moreover, the VEGF positive cells were significantly increased in mice treated with PDF based on immunohistochemistry staining, while Sal A markedly reduced the amount of VEGF positive cells. The morphologic findings were further verified by calculating the CD31 positive area per microscopic field and by absolute counting of VEGF positive cells (Figure 5B).

### GSK3β Inhibition Mediates the Effect of Sal A on Nrf2 Response and NFκB Signaling in Peritoneal Mesothelial Cells Upon PDF Injury

**Figure 6:**
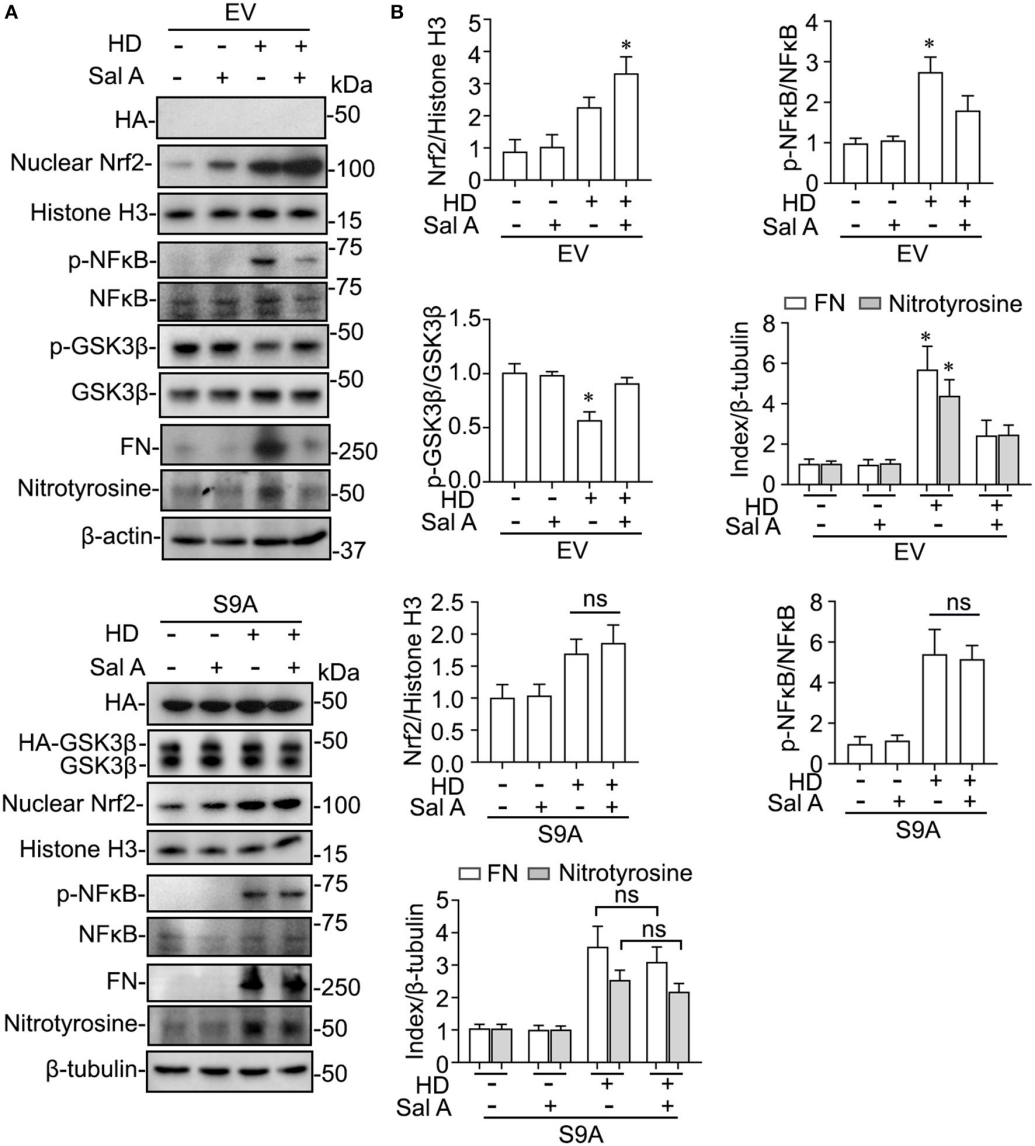
GSK3β inhibition is required for the regulatory effect of Sal A on NFkB and Nrf2 activation in cultured peritoneal mesothelial cells exposed to hypertonic dextrose. **(A)** Primary cultures of murine peritoneal mesothelial cells were transfected with the empty vector (EV) or the vector encoding the HA-conjugated constitutively active mutant of GSK3β (S9A) and then were exposed to hypertonic dextrose (HD) or vehicle treatment for 48 h. Representative immunoblots showing immunoblot analysis of collected cells for indicated molecules. **(B)** Relative abundance of indicated proteins, as normalized to the expression of histone H3, NFκB, total GSK3β, β-actin or β-tubulin based on densitometric analyses of immunoblots (*n* = 3). Data were analyzed by one-way ANOVA followed by Tukey's test. *P < 0.05 vs. other groups. ns, not significant.

Evidence suggests that GSK3β is a convergent point of the signaling cascades regulating both Nrf2 and NFκB pathways and thus is likely involved in antioxidant response and inflammation. To discern if there is a causal relationship between the above-observed inhibitory effect of Sal A treatment on PDF-elicited GSK3β hyperactivity and the beneficial effect of Sal A on oxidative injury and inflammation, primary cultures of PMC were employed and treated with medium containing hypertonic (4.25%) dextrose to model PDF-elicited peritoneal fibrosis *in vitro* (Figure 6). Reminiscent of the key features of PDF-elicited peritoneal fibrosis, hypertonic dextrose elicited considerable profibrotic changes, inflammatory reaction and oxidative damage in control empty vector-transfected PMC cultures, marked, respectively, by fibronectin overexpression, increased phosphorylation and activation of NFκB RelA/p65, and induction of nitrotyrosine, concomitant with an antioxidant self-defense as evidenced by increased nuclear expression of Nrf2. In consistency, the injurious effects of hypertonic dextrose were associated with GSK3β hyperactivity, marked by elevated GSK3β expression and reduced inhibitory phosphorylation of GSK3β at serine 9 residue. In contrast, Sal A treatment averted the GSK3β hyperactivity triggered by hypertonic dextrose. Accordingly, these injurious effects of hypertonic dextrose were substantially abolished by Sal A treatment, in parallel with an enhanced nuclear expression of Nrf2, indicative of a reinforced antioxidant response (Figure 6). In contrast, in PMC cultures expressing the constitutively active GSK3β mutant as shown by immunoblot analysis for HA, the protective effect of Sal A against the hypertonic dextrose instigated profibrotic changes, inflammatory reaction, and oxidative damage was largely blunted (Figure 6). These findings suggest that Sal A potentiates Nrf2 response and mitigates NFκB signaling *via* GSK3β inhibition.

## Discussion

Peritoneal fibrosis is a common and devastating complication in patients undergoing peritoneal dialysis, and a major cause responsible for peritoneal membrane ultrafiltration dysfunction and failure of peritoneal dialysis (1). Our present study demonstrated that Sal A, an active component of *S. miltiorrhiza*, ameliorates peritoneal fibrosis by mitigating oxidative injury and inflammation in a mouse model of peritoneal dialysis. Accumulating evidence recently suggests that *S. miltiorrhiza* exerts a unique anti-fibrotic effects in pre-clinical models of tissue fibrogenesis in a number of organ systems, including the heart (25), lung (26), liver (27), and kidney (28). However, its role in peritoneal fibrosis is unknown. To the best of knowledge, this is the first study demonstrating that the active component of *S. miltiorrhiza* Sal A possesses a potent protective effect against peritoneal fibrosis *in vivo* in mouse models of peritoneal dialysis and *in vitro* in peritoneal mesothelial cells stimulated by hypertonic dextrose.

Regardless of the original etiology, diseases in different organ systems progress *via* the final common pathways that include fibrogenesis, inflammation and oxidative injury (3). The beneficial effect of *S. miltiorrhiza* on multiple experimental disease models has been associated with improvement in these final common pathways (25–28). Nevertheless, the underlying molecular mechanisms are controversial. There is evidence suggesting that *S. miltiorrhiza* is able to intercept a number of cell signaling cascades that modify these pathologic pathways. For instance, in rat PMC exposed to high ambient glucose, *S. miltiorrhiza* injection was able to attenuate the loss of E-cadherin mRNA expression and repress the induction of mRNA expression of a-smooth muscle actin, suggesting that *S. miltiorrhiza* has an anti-fibrotic effect in peritoneal fibrosis (29). In addition, in murine models of *Gynura segetum*-induced hepatic sinusoidal obstruction syndrome, *S. miltiorrhiza* was shown to confer a hepatoprotective effect that mitigated the expression of proinflammatory mediators, like TNF-a, VCAM-1, and ICAM-1 in diseased livers (30). This protective effect of *S. miltiorrhiza* was found to be associated with reduced activity of NFκB p65 (30). This is in line with the observation by Yang et al. (31), in which administration of *S. miltiorrhiza* extract reduced myocardial infarction-induced inflammation, as evidenced by the decreased expression of proinflammatory cytokines such as IL-1β, TNF, and IL-6. In consistency, the present study also demonstrated an inhibitory effect of Sal A on NFκB activation in peritoneum of peritoneal dialysis models, associated with amelioration of inflammatory infiltration and maladaptive endothelial proliferation and angiogenesis. Apart from the anti-inflammatory property, *S. miltiorrhiza* has been noted to have a potent antioxidant activity. To this end, in rats with paracetamol-induced liver injury, *S. miltiorrhiza* was shown to significantly heighten the ratios of reduced glutathione (GSH) to oxidized glutathione (GSSG), denoting an antioxidant effect (32). In agreement, Wang et al. (33) found that treatment with extracts of *S. miltiorrhiza* increased the expression of heme-oxygenase-1, NAD(P)H quinine oxidoreductase, and Nrf2 in the injured brain tissues and resulted in a neuroprotective effect against cerebral ischemia injury in rats. Our findings here consistently suggest an antioxidant activity of *S. miltiorrhiza*, as evidenced by the reinforced Nrf2 antioxidant response and diminished expression of nitrotyrosine.

**Figure 7:**
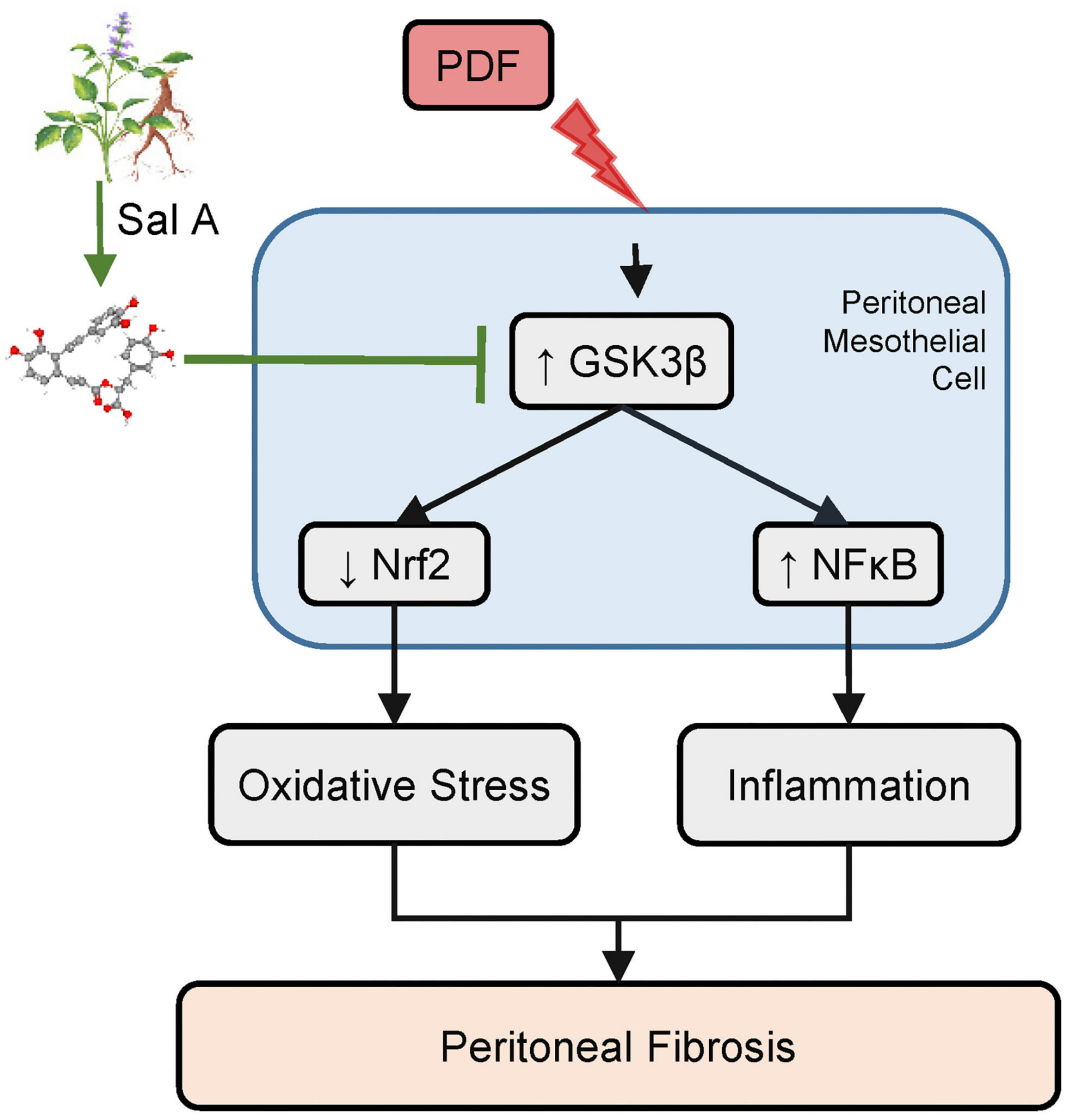
A schematic diagram depicts the molecular mechanism underlying the protective effect of Sal A, the active ingredient of *Salvia miltiorrhiza*, on peritoneal fibrosis, inflammation, and oxidative injury in murine models of peritoneal dialysis. As a key active component of *Salvia miltiorrhiza*, Sal A is able to directly bind to and inhibit GSK3β (14). GSK3β is a convergent point of myriad pathways modifying the severity of peritoneal fibrosis, including the NFκB directed inflammatory response pathway and the Nrf2 antioxidant pathway. Inhibition of GSK3β by Sal A mitigates NFκB phosphorylation and activation and reinforces Nrf2 antioxidant self-defense in peritoneal mesothelial cells and thereby attenuates inflammation, maladaptive angiogenesis and oxidative damage in peritoneum exposed to the hypertonic PDF, resulting in an improved peritoneal fibrosis.

In light of the multipronged effect of *S. miltiorrhiza* on various pathologic pathways like inflammation and oxidative stress, it is tempting to speculate that the active ingredient of *S. miltiorrhiza* that mediates the therapeutic action may possibly target some regulatory signaling transducers upstream of and common to diverse pathologic pathways. Among these regulatory signaling transducers, GSK3β has emerged as a crucial modifier of a myriad of disease processes, including inflammation, oxidative stress, fibrogenesis and tissue injury, repair and injury (21, 34, 35). GSK3β was originally identified to be a critical mediator of the insulin and glycogen biosynthesis pathway (34). Later, more and more data revealed that it is also a key kinase regulating the activity of many other signaling pathways (34), including the Wnt/β-catenin pathway, the Hedgehog signaling, the Notch pathway and cytoskeleton integrity. More recently, GSK3β has been implicated in the regulation of proinflammatory NFκB activity (36). It was found that GSK3β was sufficient and essential for NFκB RelA/p65 phosphorylation, which specifies the expression of selective NFκB target molecules, including specifically proinflammatory cytokines and mediators (37). As such, GSK3β-dicated fine-tuning of NFκB plays a key role in controlling the severity of inflammatory injury (7). In addition, Nrf2 is a cognate substrate for GSK3β (38). GSK3β-directed nuclear exclusion and degradation of Nrf2 is pivotal in switching off the self-protective antioxidant stress response after injury (39). Thus, GSK3β-directed regulation of Nrf2 represents a key mechanism of control of the magnitude and duration of the stress elicited Nrf2 antioxidant response at a delayed/late phase following insults (40). Several lines of evidence from our study suggests that *S. miltiorrhiza* may target GSK3β to exert its beneficial action in diseases. First, the active component of *S. miltiorrhiza* Sal A was able to avert GSK3β hyperactivity in peritoneal tissues exposed to PDF, concomitant with the anti-fibrotic, anti-inflammatory and antioxidant effect. In addition, the protective effect of Sal A on peritoneal fibrosis was recapitulated *in vitro* in peritoneal mesothelial cells injured with hypertonic dextrose and abolished by overexpression of the constitutively active GSK3β. Therefore, we posited that Sal A is likely to have a GSK3β inhibitory activity (Figure 7). In support of our findings, Zhang et al. (13) demonstrated that Sal A was able to protect against progressive chronic kidney injury in in 5/6 nephrectomized rats by activating the GSK3β/Nrf2 signaling pathway and inhibiting the NFκB. It is not clear if Sal A directly inhibits GSK3β activity or indirectly *via* other mediators. However, there is evidence favoring the former postulation. By using the automated high-throughput screening Kinase-Glo luminescent kinase assay platform, Paudel et al. (14) found that Sal A is an ATP-competitive inhibitor of GSK3β with an inhibitory potency comparable to standard small molecule inhibitors of GSK3β. The mechanism of action was revealed by *in silico* modeling to be the hydrophobic, π-cation, and hydrophilic interactions of Sal A at ATP and substrate sites of GSK3β. Admittedly, the present study is a pilot one with a short period in animal models. Further studies are merited to ascertain the safety and efficacy of Sal A therapy in preventing peritoneal fibrosis in patients undergoing peritoneal dialysis. Admittedly, *S. miltiorrhiza* also contains other active components, such as Tanshinone, which has been shown to be protective against peritoneal fibrosis (41). In addition, multiple other components of *S. miltiorrhiza* also have been shown to prevent peritoneal adhesions (42). In light of the molecular mechanism uncovered by the present study, it merits further studies to determine if GSK3β signaling pathway is involved in the beneficial activities of other components of *S. miltiorrhiza*.

In summary, Sal A, the active ingredient of *S. miltiorrhiza* ameliorates peritoneal fibrosis and mitigated oxidative injury and inflammation *in vivo* in a mouse model of peritoneal dialysis and *in vitro* in peritoneal mesothelial cells exposed to hypertonic dextrose. The beneficial action of Sal A is likely mediate *via* at least in part GSK3β inhibition. Our findings may pave the way for developing a novel therapy for preventing peritoneal fibrosis and ultrafiltration failure in peritoneal dialysis.

## Data Availability Statement

The raw data supporting the conclusions of this article will be made available by the authors, without undue reservation.

## Ethics Statement

The animal study was reviewed and approved by the Ethics Committee of the First Affiliated Hospital of Zhengzhou University.

## Author Contributions

PW and FZ contributed to the study design. FZ, LY, and XL carried out animal and cell culture experiments. FZ, YL, and XH performed the data analysis. FZ and PW contributed to the manuscript editing. All authors have read and agreed to the published version of the manuscript.

## Funding

This work was supported in part by the National Natural Science Foundation of China Grants 81873612 and 81770672 (to PW) and Foundation of He'nan Educational Committee Grants 20B320040 and 19B320019 (to XH). The funders had no role in the design and conduct of this study, collection and interpretation of the data, or preparation and approval of the manuscript.

## Conflict of Interest

The authors declare that the research was conducted in the absence of any commercial or financial relationships that could be construed as a potential conflict of interest.

## Publisher's Note

All claims expressed in this article are solely those of the authors and do not necessarily represent those of their affiliated organizations, or those of the publisher, the editors and the reviewers. Any product that may be evaluated in this article, or claim that may be made by its manufacturer, is not guaranteed or endorsed by the publisher.
